# Pregnancy associated cardiomyopathy in 2 cancer survivors with history of anthracycline use^☆^

**DOI:** 10.1016/j.ahjo.2025.100539

**Published:** 2025-04-04

**Authors:** Ramy Zughul, Navya Akula, Isaac Rhea, Michael Zacharias, Heather Blume, Chantal ElAmm

**Affiliations:** Department of Cardiology, University Hospitals, Case Western Reserve University, Advanced Heart Failure and Transplant Cardiology, Harrington Heart and Vascular Institute, Cleveland, OH, USA

**Keywords:** Cardio-obstetrics, Heart failure, Pregnancy associated cardiomyopathy, Anthracycline cardio toxicity

## Abstract

Cardiac complications in childhood cancer survivors are increasingly recognized, with radiotherapy and anthracycline exposure being major culprits. The incidence of recurrent cardiomyopathy in this population is significant, while the incidence of new-onset cardiomyopathy remains rare. This case series presents two cases of pregnancy-associated cardiomyopathy with a history of anthracycline use.

## 1 Background

Cardiac complications in childhood cancer survivors is becoming more recognized as new therapies emerge and healthcare improves. Exposure to radiation therapy and anthracycline are considered primary culprits. Other cardiac disorders that can develop include conduction system disease, ischemic heart disease or valvular dysfunction. Different factors influence the extent of cardiomyopathy or tissue damage including radiation dose and field, cumulative anthracycline dose, plus other host related factors [[1], [2], [3], [4]]. Pregnancy physiologic and hemodynamic stresses on the cardiovascular system and is found to be associated with development of or worsening of cardiomyopathy among young cancer survivors. However, limited research suggests that while incidence of recurrent cardiomyopathy is significant and reached up to 19 % in a recent cohort of patients with history of anthracycline and/or radiation associated cardiomyopathy [1]. The incidence of new onset cardiomyopathy in those with remained rare in that cohort (0.3 %), and this is substantiated by previous reports as well [5,6]. Anthracycline use has been reported in as many as one third of cancer survivors in the United States [7] and therefore should still be considered as a cause of cardiomyopathy in this population. In this case series, we share our experience at the cardio-obstetrics clinic with 2 cases of pregnancy associated cardiomyopathy in the setting of history of anthracycline use.

## 2 Case 1

### 2.1 History of presentation

The patient presents has a history of anthracycline-induced cardiotoxicity with mildly reduced EF was referred to the Cardio-Oncology clinic after becoming pregnant at 16-week gestation.

### 2.2 Past medical history

A 26-year-old Gravida 1 Para 0 Female who has a history of asthma, obesity (BMI 42), and Hodgkin's disease treated 13 years prior with modified BEACOPP and mantle field.

Her previous cancer treatment included bleomycin, etoposide, doxorubicin IV 240 mg/m2, cyclophosphamide, vincristine, procarbazine, prednisone, and vinblastine. She also received mantle radiation, total of 36 Gy.

### 2.3 Investigations

Her most recent transthoracic echocardiogram prior to presentation showed mildly diminished left ventricular (LV) systolic function based on a shortening fraction of 26 % (normal range 25–45 %). The LV appeared mildly dilated, with limited ability to accurately obtain an LV ejection fraction (LVEF). LV global longitudinal strain (GLS) was abnormal at −15 % (normal range −18 to −24 %). The patient had no significant valvular disease. Qualitatively right ventricular size was normal, with normal systolic function.

NT-proBNP at time of visit was 45 pg/mL (reference range 0–100 pg/mL).

### 2.4 Management plan

With a goal of minimal pharmacologic treatment during pregnancy, no cardiac medications were added, and she had serial monitoring with echocardiograms every 2 months.

### 2.5 Clinical course

**Fig. 1:**
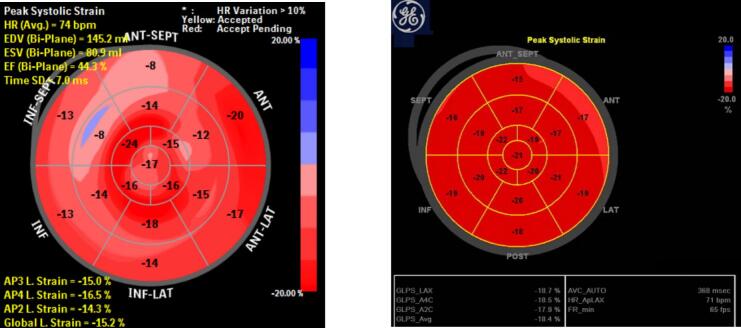
Global longitudinal strain at 2 weeks (GLS -15.2 %) and at 25 weeks (GLS -18.4 %), GLS normal range: −18 to −24 %. HR: heart rate. EDV: end diastolic volume. ESV: end systolic volume. EF: Ejection fration. AP: apical.

The patient's cardiac function improved with most recent LVEF prior to delivery of 55 %, and LV GLS of −18.4 % (Fig. 1). Follow-up cardiac MRI showed left ventricle is normal in size and shape with mild global systolic dysfunction and no segmental wall motion abnormalities.

During this time, she experienced multiple bouts of palpitations; subsequently confirmed to be SVT with maximum rate of 190 on outpatient monitor, successfully treated with low dose metoprolol tartrate.

She successfully delivered at 38 weeks via Cesarean due to failed induction of labor, with no subsequent cardiac or obstetric complications.

### 2.6 Follow-up

After delivery she had multiple bouts of asthma exacerbations and was transitioned to metoprolol succinate 25 mg daily and ACE-Inhibitor (ramipril) 10 mg daily. The patient has done well since with only NYHA class 1 symptoms.

## 3 Case 2

### 3.1 History of presenting illness

27-year-old patient with history of Hodgkin's lymphoma presents to the emergency room at 9 weeks gestation, complaining of shortness of breath and palpitations.

### 3.2 Past medical history

A 27-year-old female Gravida 2 Para 1 with history significant for Hodgkin's lymphoma treated with R-CHOP x6, completed 5 months prior, presented to the emergency department at 9 weeks gestation with shortness of breath and palpitations. In the ED she was diagnosed with miscarriage of twins.

### 3.3 Investigations

Workup revealed a new drop in her left ventricular ejection fraction (LVEF) of 15 %. Her previous serial echocardiograms during chemotherapy treatment did not show reduction in EF until that visit.

### 3.4 Management

After following with cardiology, she was started on spironolactone, carvedilol, and ramipril; she did not tolerate sacubitril/valsartan due to hypotension. Ivabradine was also introduced due to the inability to achieve a sufficient carvedilol dose for heart rate reduction, owing to the patient's hypotension.

### 3.5 Clinical course

At 3 month follow up her LVEF improved to 40 % and continued to improve to 55 % at 7 months.

### 3.6 Follow-up

Three months later, the patient presented to the cardio-obstetrics clinic with another pregnancy, despite using a contraceptive medroxyprogesterone acetate (Depo-Provera®). EKG showed normal sinus rhythm at rate 72 and nonspecific T wave changes. Her ramipril and spironolactone were held due to their known adverse effects in pregnancy. Unfortunately, she had another miscarriage a week later.

A subsequent pregnancy was successful with EF of 55 % maintained during pregnancy with all GDMT discontinued aside from metoprolol.

The patient continued metoprolol monotherapy due to wishing to breastfeed. Subsequently EF declined to 35 % and she became symptomatic, however symptoms improved after weaning to bottle feeding, restarting previous GDMT, and adding diuretics. Despite maximal therapy, she had persistent NYHA class 2 symptoms.

## 4 Discussion

Pregnancy-associated cardiomyopathy is a rare occurrence among childhood cancer survivors and can precipitate cardiac dysfunction related to previous chemotherapy or radiation exposure. Retrospective cohorts have identified a higher incidence of this condition in patients who had evidence of cardiac dysfunction prior to becoming pregnant, even in those with recovered EF before pregnancy [[1], [2], [3], [4]]. Conversely, incidence is significantly lower in patients with prior normal cardiac function. However, it is important to be aware of patients who show evidence of cardiac dysfunction but are asymptomatic, as they may have subclinical cardiomyopathy with an increased risk of recurrent or worsening dysfunction [1]. LV global longitudinal strain via speckle tracking, has emerged as a useful tool for early detection of cardiac dysfunction and remodeling and can be used in these patients [7].

Evidence of cardiotoxicity is usually seen in higher cumulative anthracycline doses (in excess of 240 mg/m^2^) [1]. Several mechanisms of anthracycline toxicity have been proposed, including myocardial toxicity due to complexing with iron causing oxygen-free radicals, for which reason iron chelators and free radical scavengers have been proposed as potential treatment options (e.g. dexrazoxane). Other mechanisms include activation of topoisomerase II beta isoform in cardiac myocytes causing double-stranded DNA breaks and cell death. Dexrazoxane (EDTA Derivative) showed effects independent of iron chelation and free radical scavenging by competitive binding to this enzyme and is the only therapy that was shown in randomized trials to improve cardiomyopathy related to anthracyclines, however due to concerns about its potential reduction of desired effects on cancer cells as well, its use remains limited [1]. Other mechanisms include anthracycline interaction with apoptotic pathways (like p53) and angiogenic pathways (like VEGF and c-kit+ endothelial progenitor cells) disrupting angiogenic balance and causing myocardial growth restriction and increased vascular stiffness [7]. Other chemotherapy agents cause cardiomyopathy through different mechanisms, such as cyclophosphamide which is suspected to cause myocarditis [8].

Guideline-directed medical therapy (GDMT) for heart failure remains the primary approach for cardiomyopathy in cancer survivors and is also the preferred treatment for pregnancy-associated cardiomyopathy in these patients. However, attention should be given to the limitations of using certain medications during pregnancy and breastfeeding [7]. This case report demonstrates an approach to the management and monitoring of two patients within this category, as well as the outcomes observed during follow-up. However, given the anticipated increase in the prevalence of this disorder, it is essential to obtain a larger sample size, potentially from multicenter registries, to thoroughly study this patient population and their long-term responses to therapy. Furthermore, a more robust pharmacologic investigation of the benefit-risk profile of guideline-directed medical therapy (GDMT) in pregnant patients is warranted. Regarding the mode of delivery for women with dilated cardiomyopathy, vaginal delivery is generally recommended due to the associated lower blood loss and better hemodynamic stability compared to other delivery methods [9]. However, a multidisciplinary approach should be emphasized, particularly for patients with compromised cardiac function or those at risk for prolonged labor. Pre-planning should include considerations for potential labor progression failure and clear indications for assisted or operative interventions when necessary.

### 4.1 Conclusion

Chemotherapy and radiation associated cardiomyopathy should be considered in pregnant patient presenting with cardiomyopathy. Patients with evidence of clinical or subclinical cardiac dysfunction have higher risk of worsening or recurrent dysfunction, these patients should be followed with serial echocardiograms. Mainstay treatment is GDMT, while being aware of limitations during pregnancy or breast-feeding period.

### 4.2 Learning objectives

1. Identify chemotherapy-induced cardiomyopathy as a potential cause or precipitating factor of cardiomyopathy in cancer survivors during pregnancy.
2. Understand the individualized monitoring approaches for pregnant patients with a history of anthracycline use, which may include careful monitoring of cardiac function through regular echocardiograms and biomarker assessments (such as NT-proBNP), and more advanced techniques such as LV global longitudinal strain.
3. Management strategies should focus on cautious implementation of pharmacologic interventions during pregnancy while ensuring optimal cardiac function. For patients exhibiting signs of worsening cardiac function, a multidisciplinary care approach involving cardiology, obstetrics, and oncology may be necessary to tailor interventions, such as adjusting medications or implementing supportive therapies, thereby enhancing maternal and fetal outcomes.
4. Emphasize the importance of referral to a cardio-obstetrics clinic for enhanced monitoring and management of cardiac complications during pregnancy.

## CRediT authorship contribution statement

**Ramy Zughul:** Writing – original draft. **Navya Akula:** Writing – original draft. **Isaac Rhea:** Supervision. **Michael Zacharias:** Writing – review & editing, Supervision. **Heather Blume:** Writing – original draft, Conceptualization. **Chantal ElAmm:** Writing – review & editing, Supervision.

## Funding

This research did not receive any specific grant from funding agencies in the public, commercial, or not-for-profit sectors.

## Declaration of competing interest

No known competing financial interests or personal relationships that influence the work reported in this paper.
